# Etiology of Coronary Reintervention After Coronary Artery Bypass Surgery

**DOI:** 10.3390/jcdd13010020

**Published:** 2025-12-31

**Authors:** Ikram Achbar, De Qing F. N. Görtzen, Joost F. J. ter Woorst, Koen Teeuwen, Pim A. L. Tonino, Ferdi Akca

**Affiliations:** 1 Department of Cardiothoracic Surgery, Catharina Hospital, 5623 EJ Eindhoven, The Netherlands; 2 Department of Interventional Cardiology, Catharina Hospital, 5623 EJ Eindhoven, The Netherlands; 3 Department of Biomedical Engineering, Technical University Eindhoven, 5631 BN Eindhoven, The Netherlands

**Keywords:** coronary artery bypass grafting, reintervention, incomplete revascularization, graft failure, progression of disease

## Abstract

(1) Background: Coronary artery bypass grafting (CABG) reduces the risk of target vessel revascularization compared to percutaneous coronary intervention (PCI), yet coronary reintervention may still occur. This study aims to evaluate the incidence and underlying etiology of reintervention after CABG. (2) Methods: A single-center retrospective cohort study of all patients undergoing isolated CABG (January 2016–December 2021) was performed. Surgical or percutaneous reinterventions were analyzed until December 2022 using institutional data linked to the Netherlands Heart Registration (NHR) and chart review. (3) Results: Amongst 4814 patients, 8.7% (n = 418) underwent coronary reintervention during a median 4.5 [3.8–4.8] year follow-up. Causes of reintervention included graft failure (64.6%), progression of coronary artery disease (20.3%), incomplete revascularization (10.5%), or combined factors (4.1%). Mortality did not differ significantly between reintervention and non-reintervention groups (10.8% vs. 7.9%, *p* = 0.095). Multivariable analysis identified diabetes (HR 1.02, 95% CI 1.00–1.04, *p* = 0.011), single arterial graft (HR 2.26, 95% CI 1.31–3.91, *p* = 0.003), and ventilation > 24 h (HR 4.61, 95% CI 1.85–11.51, *p* = 0.001) as independent risk factors for coronary reintervention. (4) Conclusions: After CABG, 8.7% of patients underwent coronary reintervention at mid-term follow-up. Graft failure was the predominant etiology, followed by coronary artery disease progression. Overall survival did not differ between patients with or without reintervention.

## 1. Introduction

A key advantage of coronary artery bypass grafting (CABG) in patients with three-vessel coronary artery disease (CAD) is the lower risk of target vessel revascularization compared to percutaneous coronary intervention (PCI) [1,2,3]. Despite the favorable target vessel revascularization incidence of CABG, patients might still need a subsequent coronary procedure during their life. Previous studies reported incidences of coronary reintervention after CABG between 5.4% and 13.7% at 5-year follow-up [2,3,4]. However, the underlying etiology requiring reintervention after CABG remains underreported, whereas such knowledge can further improve the field of coronary surgery.

In clinical practice, postoperative angina symptoms in postoperative patients may indicate the need for coronary reintervention. Multiple reasons may underlie the need for coronary reintervention, such as (1) progression of coronary artery disease in arteries that did not require revascularization at the initial CABG procedure, (2) failure of (arterial and/or venous) bypass graft conduits, (3) incomplete revascularization during the initial CABG procedure, and (4) a combination of the previous three reasons [5].

In this study, we aimed to evaluate the incidence and etiology of coronary reintervention after CABG. The results of such analysis can be of importance in understanding the underlying pathophysiology requiring subsequent revascularization after CABG and as a consequence, might be of help in improving outcomes after CABG.

## 2. Materials and Methods

### 2.1. Patient Inclusion

A single-center retrospective cohort study was conducted. Patients who underwent initial isolated CABG (both on-pump and off-pump) between January 2016 and December 2021 were included. The data were accessed on 25 August 2023. The authors had access to information that could identify individual participants during and after data collection; however, the data were anonymized prior to analysis. Patients with angina symptoms and/or patients with positive ischemia detection underwent reintervention. In this cohort all surgical or percutaneous reintervention procedures until December 2022 were analyzed. Only isolated CABG patients were included; concomitant valvular disease was excluded. Patients who underwent a redo-operation, salvage surgery, or minimally invasive bypass grafting as part of a hybrid revascularization strategy were also excluded. The patients for whom, during the follow-up period, no coronary angiogram or report of the angiogram could be obtained were excluded from analysis. Furthermore, patients for whom no medical records were available during follow-up were also excluded from analysis.

### 2.2. Data Collection and Parameters

Data were retrieved from our center’s database, which is linked to the Netherlands Heart Registration, a national quality registry including all invasive cardiac interventions, electrophysiologic procedures, and cardiac surgeries [6]. This registry collects baseline, procedural, and outcome data of all procedures. Reinterventions performed in the cohort in other hospitals were identified through the Netherlands Heart Registration and were subsequently verified in our database. Additional data were retrieved through chart review, including Heart Team recommendations by myocardial territory, surgical details such as graft configurations or reasons for incomplete revascularization, postoperative coronary angiography (CAG) images, and reports to identify the reason for coronary reintervention.

The baseline characteristics retrieved from the database were age, gender, height, weight, history of cerebrovascular accident (CVA), atrial fibrillation, previous PCI, chronic lung disease, peripheral artery disease, neurological dysfunction, poor mobility, previous cardiac surgery, creatine level, dialysis, diabetes, Canadian Cardiovascular Society (CCS) class IV, unstable angina, left ventricle ejection fraction (LVEF), recent myocardial infarction, pulmonary artery pressure, New York Heart Association (NYHA) class, urgency of operation, and EuroSCORE II. The extent of coronary artery disease (CAD) and left main disease for the reintervention group was also reported. The extent of coronary artery disease and left main disease was classified according to the 2024 ESC guidelines for the management of chronic coronary syndromes and the 2023 ESC Guidelines for the management of acute coronary syndromes [7,8,9].

Perioperative parameters included the extent of coronary artery disease, extracorporeal circulation (ECC) details (cannulation method, duration, and aortic cross-clamp time), surgical approach, the total number of anastomoses (both arterial and venous) and graft usage (left internal mammary artery (LIMA), right internal mammary artery (RIMA), radial artery, and venous grafts).

Preoperative laboratory values (hemoglobin and hematocrit) and blood product administration (red blood cells, plasma, and platelets) during hospital stay were collected. Outcome measures included in-hospital mortality and overall mortality, reoperation during admission (including re-exploration for bleeding), duration of hospital stay, ventilation exceeding 24 h, Intensive Care Unit (ICU) readmission, and details of reintervention, including redo surgery or PCI with corresponding dates.

For the reintervention group, the medication at discharge was manually extracted from the chart review. Medications included antihyperlipidemic medications, anticoagulation medications, Angiotensin Converting Enzyme Angiotensin Receptor Blocker (ACE-inhibitors/ARB), beta-blockers, calcium channel antagonists, and diuretics.

### 2.3. Endpoints Definitions

The primary endpoint is the incidence and etiology of coronary reintervention after CABG, categorized as (1) progression of coronary artery disease in arteries that did not require revascularization at the initial CABG procedure, (2) bypass graft failure, and (3) incomplete revascularization during initial CABG. The secondary endpoint is the survival rate after the initial CABG procedure.

### 2.4. Identification of Etiology for Reintervention

Assessment of the underlying etiology for reintervention was performed by analyzing Heart Team recommendations, postoperative CAG findings, and subsequent intervention reports. If the patient underwent a reintervention in a coronary artery not significantly diseased (either visually (<50% stenosis grade) or by fractional flow reserve measurements (>0.80)) and therefore not revascularized during the initial CABG procedure, this was classified as progression of coronary artery disease. When a patient had a reintervention in a myocardial territory with a dysfunctional bypass graft (defined as FitzGibbon B or O) this was classified as graft failure [10]. Incomplete revascularization was defined as the absence of grafting in a myocardial territory that was initially designated for revascularization by the Heart Team and subsequently became the reason for reintervention.

### 2.5. Statistical Analysis

JASP 0.18.1.0 (Universiteit van Amsterdam, Amsterdam, The Netherlands) is utilized to extract descriptives, box plots, and Q-Q plots for outliers per continuous variable. Categorical variables are presented as frequency and percentage. Continuous variables are presented as mean and standard deviation (SD) if normally distributed, and median and interquartile range (IQR) if nonnormally distributed as per graphical analysis through the Q-Q-plots. Furthermore, to compare the data, the Mann–Whitney U test, the chi-square test, or Student T-test is performed using RStudio version 4.4.0 (R Core Team, Boston, MA, USA). A *p*-value < 0.05 is considered statistically significant. The Kaplan–Meier survival curves are plotted using RStudio using the packages “*haven*”, “*survival*”, and “*survminer*”. Additionally, baseline and perioperative characteristics are analyzed using univariable and multivariable Cox regression models (including variables with a univariable *p* < 0.1) to identify independent factors contributing to reintervention.

## 3. Results

### 3.1. Patient Demographics

**Figure 1:**
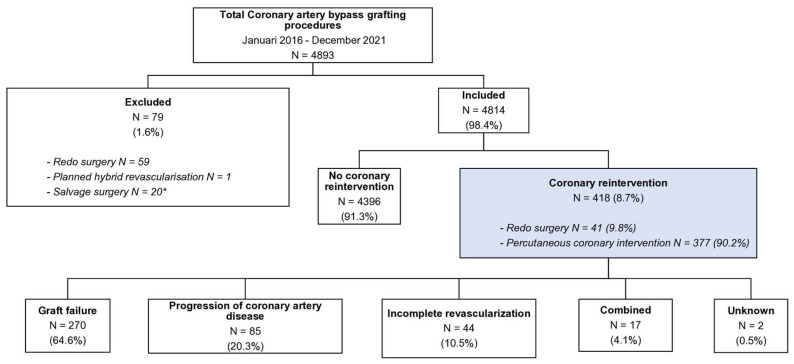
Study flow diagram. * Including one who required redo surgery.

During the study period, a total of 4893 patients underwent initial isolated CABG at our institution. Of these, 79 patients underwent a redo-operation (n = 59), hybrid revascularization (n = 1), or salvage surgery (n = 20) and were excluded. Consequently, 4814 patients were included in the final analysis (Figure 1). In 8.7% (n = 418) of patients a reintervention was required after CABG within a median follow-up period of 4.5 [3.8–4.8] years.

In Table 1, the baseline characteristics are displayed for the ‘*overall group*’, ‘*no reintervention group*’, and ‘*reintervention group*’. Compared to the ‘*no reintervention group*’, the ‘*reintervention group*’ had a significantly lower proportion of men (76.6% vs. 82.3%, *p* = 0.002), prior PCI was significantly more common (34.0% vs. 26.9%, *p* = 0.002), as was the incidence of peripheral vascular disease (18.2% vs. 13.3%, *p* = 0.019). Recent myocardial infarction was less common in the reintervention group (34.7% vs. 39.9%, *p* < 0.001). Other variables, including age, unstable angina, LVEF, chronic lung disease, atrial fibrillation, level of urgency, EuroSCORE II, and renal function, did not show statistically significant differences between the two groups.

In Table 2, the perioperative and postoperative characteristics of both groups are presented. Overall, our CABG population received a single arterial plus saphenous vein graft (SVG) in 85.5% and total arterial revascularization in 8.1%. Procedures were performed without cardiopulmonary bypass (off-pump) in 23% of patients.

In Table 3, the medication at discharge of the reintervention group is displayed. At discharge, 78.2% of the patients had statins, 41.4% acetylsalicylic acid, 59.8% ACE-inhibitors/ARBs, 90.4% beta-blockers, 24.2% calcium channel blockers, and 76.8% diuretics. A sub-analysis of discharge medication in the reintervention group revealed that 92.1% of total arterial revascularizations and 100% of those receiving radial artery grafts were prescribed calcium channel blockers at discharge. Notably, two patients did not receive anticoagulation therapy at discharge. For 14 patients, discharge medication data were unavailable due to in-hospital death or lost to follow-up.

### 3.2. Coronary Reintervention

**Figure 2:**
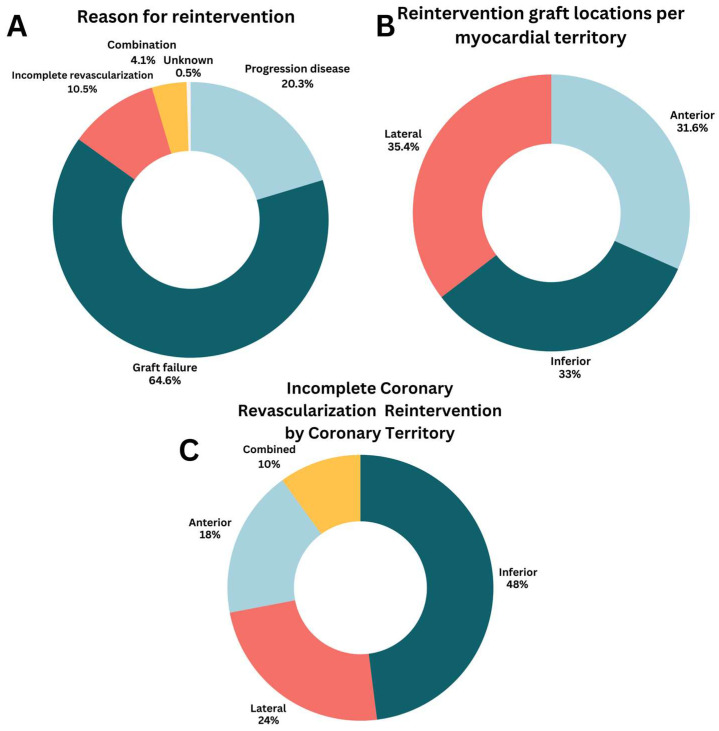
Characteristics of coronary reintervention. (**A**) Reason for reintervention; (**B**) Reintervention graft locations per myocardial territory; (**C**) Incomplete coronary revascularization reintervention by coronary territory.

In all patients who required reintervention (n = 418), a PCI was performed in 90.4% and a redo CABG in 9.6% (Table 4). The most frequent underlying etiology for reintervention was graft failure (64.6%), followed by progression of CAD (20.3%), incomplete revascularization (10.5%), a combination of the previously mentioned etiology (4.1%), and unknown etiology (0.5%) (Figure 2A). Graft failure leading to coronary reintervention occurred in 2.4% of all LIMA grafts, 3.3% of RIMA grafts, 4.4% of SVGs, and 6.1% of radial artery grafts. All myocardial territories were equally at risk for reintervention (Figure 2B).

When complete revascularization could not be achieved during the initial CABG procedure, this mostly involved the inferior wall (48%), followed by the lateral wall (24%), and anterior wall (18%) (Figure 2C). The primary reason for incomplete revascularization was an inadequate diameter/quality of the coronary target (n = 27). Other less frequent reasons were lack of graft material (n = 2) or a porcelain aorta (n = 3). In two cases (0.5%), the reason for incomplete revascularization was unknown.

### 3.3. Overall Mortality

**Figure 3:**
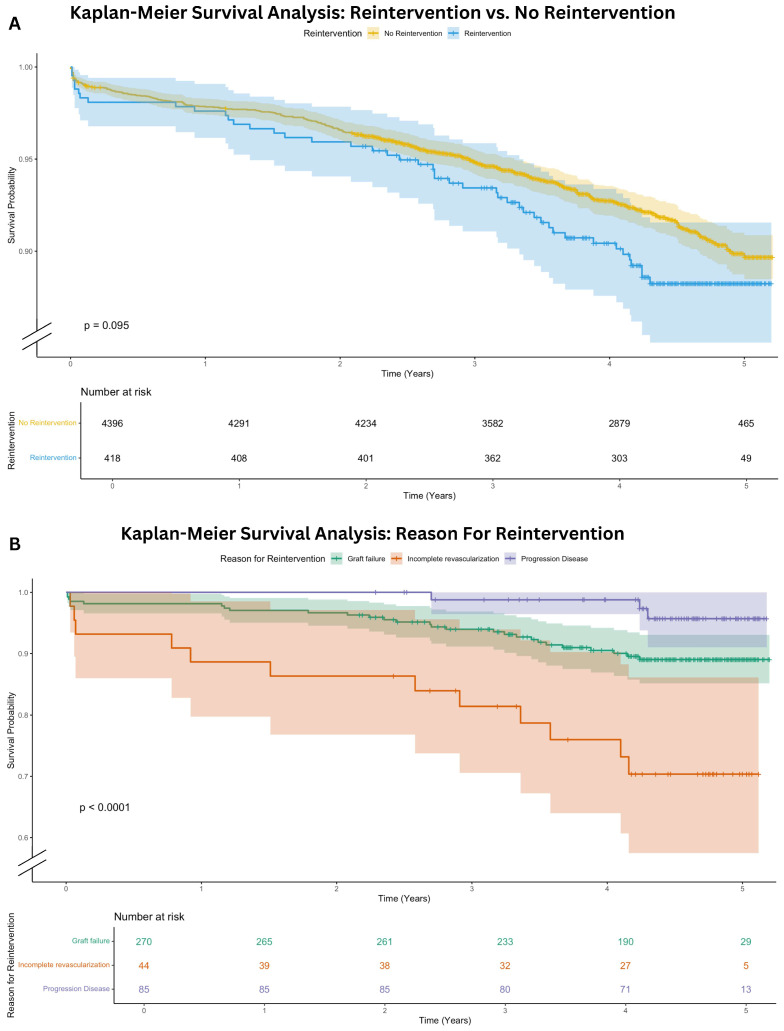
Kaplan–Meier Survival Analysis. (**A**) Kaplan-Meier survival analysis: reintervention versus no reintervention; (**B**) Kaplan-Meier survival analysis: reason for reintervention.

After a median follow-up of 4.5 [3.8–4.8] years, the overall mortality was comparable for patients with reintervention (10.8%) to patients without reintervention (7.9%, *p* = 0.095). A Kaplan–Meier survival analysis including a log-rank test is shown in Figure 3A. In Figure 3B the postoperative overall survival is presented for each specific subgroup of reintervention etiology (graft failure, progression of CAD, incomplete revascularization). Patients with incomplete revascularization had the lowest overall survival, followed by graft failure and progression of CAD (log-rank, *p* < 0.001).

### 3.4. Regression Analysis

Table 5 presents the univariable and multivariable Cox regression analysis for the occurrence of coronary reintervention. The multivariable analysis demonstrated that diabetes (Hazard Ratio 1.02, 95% CI 1.00–1.04, *p* = 0.011), single arterial graft (Hazard Ratio 2.26, 95% CI 1.31–3.91, *p* = 0.003), and ventilation > 24 h (Hazard Ratio 4.61, 95% CI 1.85–11.51, *p* = 0.001) were identified as independent risk factors for coronary reintervention. A factor that was independently associated with a lower risk of reintervention was weight (Hazard Ratio 0.98, 95% CI 0.97–1.00, *p* = 0.044).

## 4. Discussion

In this single-center retrospective cohort study of 4814 patients undergoing CABG, a coronary reintervention occurred in 8.7% of patients after a median follow-up of 4.5 [3.8–4.8] years. In 90.4%, these reinterventions are PCI, and 9.6% required surgical reintervention. To our knowledge, this is the first contemporary study to analyze the etiology of reintervention after CABG. Our data reveal that graft failure is the predominant cause (64.6%), followed by native CAD progression (20.3%), incomplete revascularization (10.5%), and a combination of these factors (4.1%) (Figure 2A). Graft failure leading to reintervention occurred most frequently in the radial artery conduit (Table 4). Overall, there was no significant difference in mortality for patients with a reintervention (10.8%) compared to patients without a reintervention (7.9%, *p* = 0.095). However, patients with incomplete revascularization have the worst overall survival within the reintervention group, as illustrated in Figure 3B.

The incidence of reintervention found in our study (8.7%) corresponds with previous studies, with an incidence between 5.4% and 13.7% at 5 years follow-up [2,4].

In our cohort, 90.4% of patients requiring reintervention underwent PCI compared to 9.6% requiring redo CABG. Faisaluddin et al. similarly reported 95.4% PCI versus 4.6% redo CABG after acute coronary syndrome in patients with a prior history of CABG [11]. No recent studies have systematically evaluated the distribution of PCI versus redo CABG across elective and acute coronary syndrome settings following initial CABG.

### 4.1. Reason for Reintervention

In our study, graft failure was the most frequent etiology for coronary reintervention after CABG. Previous studies report short and long-term graft failure rates of 2.9–8.5% for the LIMA and 15–39.8% for SVG, and 4.0–12.9% for the radial artery [12,13,14,15]. In our overall population, 5.6% received a reintervention because of graft failure, which is lower than the reported graft failure rates detected by CAG/CT bypass by protocol in other studies. However, only clinically driven revascularization is analyzed in our study and the rate of asymptomatic graft failure remains unknown. After graft failure, progression of native CAD was the most frequent cause of reintervention (1.8% of the overall population). Multiple studies have demonstrated progression of native CAD after an intervention (CABG or PCI), implying that future reintervention is not always avoidable for this systemic disease. Lifestyle management and medical therapy are key factors in lowering the risk of progression to clinically important CAD after intervention [9,16,17,18].

Graft configuration did not show a significant difference in reintervention incidence after multivariable analysis at median follow-up of 4.5 [3.8–4.8] years. Total arterial grafting, when performed infrequently, can be technically challenging and is associated with an evident volume–outcome relationship [19]. Nevertheless, the benefit of total arterial grafting as reported in the literature only becomes apparent during long-term follow-up [9,15,18,20,21].

During the study period, only 8.1% of the patients underwent total arterial revascularization, and the radial artery was used in 6.1% of patients. In our center, complete arterial grafting was not routinely performed during the study period, resulting in a limited use of the radial artery. In more recent years, however, there has been an increasing trend toward both complete arterial revascularization and the use of the radial artery in our center. According to published evidence, revascularization with the radial artery is associated with lower rates of graft occlusion and improved survival [20].

Furthermore, the specific coronary anatomy and degree of coronary stenosis, which are known to influence radial artery patency, were not available in our dataset [15,22]. Likewise, data on vessel quality and operator technique were missing, limiting our ability to adjust for these important confounders. While our findings appear to contradict some of the literature reporting superior long-term patency of radial grafts [20], this discrepancy likely reflects the influence of institutional practice, data regarding target vessel stenosis, and differences in follow-up duration.

Medication data at discharge were only available for the reintervention cohort (Table 3). Most patients undergoing total arterial revascularization received calcium channel blockers, and this was the case for 100% of those with radial artery grafts. Although prior studies have associated calcium channel blocker therapy with improved outcomes in radial artery conduit use, the absence of comparable data in the non-reintervention group limits interpretation; therefore, any potential association should be interpreted with caution [23].

### 4.2. Predictors for Coronary Reintervention

Diabetes was identified as an independent risk factor for reintervention (Hazard Ratio 1.02, 95% CI 1.00–1.04, *p* = 0.011) (Table 5). Given that diabetes is a well-established risk factor for atherosclerosis [24], it is also associated with an increased risk of reintervention [2]. This may explain the higher Hazard Ratio observed in this study.

Furthermore, single arterial grafting was found to be an independent risk factor for reintervention (Hazard Ratio 2.26, 95% CI 1.31–3.91, *p* = 0.003) (Table 5). A possible explanation for this finding is that patients with extensive CAD may receive only a LIMA graft to the left anterior descending artery (LAD) when the remaining vessels are technically not suitable for bypass surgery. Consequently, these patients might have a higher likelihood of requiring subsequent PCI for the untreated vessels due to incomplete revascularization. However, this remains uncertain, as the present study did not specifically assess whether incomplete revascularization was more prevalent in the single arterial grafting group. In contrast to previous studies identifying female sex as a predictor of coronary reintervention, our analysis did not demonstrate a significant association. This discrepancy may be attributed to the relatively small sample size of our cohort, potentially limiting statistical power [25].

Other perioperative complications such as prolonged ventilation time (Hazard Ratio 4.61, 95% CI 1.85–11.51, *p* = 0.001), and readmission to ICU were also found to be independent risk factors for reintervention (Table 5). Readmission to the ICU could be due to acute ischemia, graft dysfunction, or overall poor post-operative condition explaining the increased risk for reintervention. Moreover, patients with extensive CAD might represent a more critically ill population, potentially associated with a higher risk of prolonged ventilation time and readmission to the ICU [26,27].

In the reintervention group, it is observed that there is a significantly higher percentage of off-pump procedures (22.4% vs. 29.7%, *p* = 0.001). However, caution should be taken to attribute the need for reintervention solely to the increased incidence of off-pump surgery. Multiple studies over the years have shown that off-pump bypass surgery (OPCAB) does not negatively impact the long-term outcomes post-surgery and might even improve outcome using an-aortic off-pump techniques [28,29,30,31,32].

### 4.3. Effect of Reintervention on Mortality

The difference in repeat revascularization and overall mortality between CABG and PCI has been previously studied [33,34,35]. In a study by Wang et al., they report comparable overall mortality between reintervention and no reintervention in a pooled cohort of CABG and PCI revascularizations [36]. In our results, we similarly observed no significant difference in mortality between both groups.

In the Kaplan–Meier analyses of both the cumulative incidence to reintervention and the overall survival analysis of the reintervention group, we observed that the incomplete revascularization group had the highest mortality, congruent to the established literature [34]. The reasons for incomplete revascularization are multifactorial, but mostly due to the technical inability to create a bypass graft. Probably these patients have more extensive CAD with anatomical complexities such as severely calcified or small-diameter vessels that make complete surgical revascularization technically challenging or even impossible. The Syntax score was unfortunately not available for assessment of CAD extent. These limitations could indicate a more advanced stage of coronary pathology, with consequently a more impaired prognosis [26,27].

### 4.4. Limitations

One of the limitations of this study is its retrospective, single-center design. Furthermore, reinterventions were performed based on the presence of clinical symptoms, without a standardized approach for assessing ischemia. The clinical outcomes of the reinterventions were not collected, thereby limiting our ability to assess the effects of reinterventions. Additionally, the data on graft failure and progression of disease are only available for the reintervention group. This means that the exact percentage of graft failure and progression of disease remains unknown.

At discharge, 99.5% of patients in the reintervention cohort received aspirin following surgery; however, data on dual antithrombotic therapy or other pharmacotherapy (e.g., DOAC, vitamin K antagonists, antihypertensive, or antihyperlipidemic therapy) were not available for the entire cohort except for the reintervention group; therefore, any potential association should be interpreted with caution. Medication adherence was also not evaluated at reintervention. And the data was only available for the primary discharge letter.

Baseline cholesterol levels were not reported, and laboratory follow-up data were lacking. Finally, sex-specific differences in outcomes could only be assessed in the reintervention cohort, as reliable outcome and follow-up data were available for these patients. In contrast, data from the non-reintervention cohort were incomplete, preventing analysis of potential sex-based differences in outcomes or patient and surgical characteristics.

## 5. Conclusions

After CABG, coronary reintervention occurs in 8.9% of patients after a median follow-up of 4.5 [3.8–4.8] years. The most frequent underlying etiology of reintervention is graft failure (64.6%), followed by progression of CAD (20.3%) and incomplete revascularization (10.5%). Overall, the occurrence of postoperative reintervention does not affect mid-term survival. However, incomplete surgical revascularization is associated with an increased risk of mortality.

## Figures and Tables

**Table 1:** Baseline Characteristics.

	Overall (N = 4814)	No Reintervention (N = 4396)	Reintervention (N = 418)	*p*-Value
Sex (men)	3961 (82.3)	3641 (82.3)	320 (76.6)	**0.002**
Age (year)	66.5 ± 9.2	66.5 ± 9.2	65.7 ± 9.3	0.097
Height (cm)	173.2 ± 14.0	173.2 ± 14.5	173.4 ± 9.3	0.508
Weight (kg)	83.2 ± 15.1	83.1 ± 13.9	83.5 ± 13.9	0.601
Diabetes				0.715
Diabetes no treatment	22 (0.5)	19 (0.4)	3 (0.7)	
Diabetes diet	75 (1.6)	68 (1.5)	7 (1.7)	
Diabetes oral medication	592 (12.3)	540 (12.3)	52 (12.4)	
Diabetes insulin	489 (10.2)	438 (10.0)	51 (12.2)	
Peripheral vascular disease	659 (13.7)	583 (13.3)	76 (18.2)	**0.019**
Atrial fibrillation				0.796
Paroxysmal atrial fibrillation	287 (93.6)	264 (6.0)	23 (5.5)	
Non-paroxysmal atrial fibrillation	15 (0.3)	14 (0.3)	1 (0.2)	
Unstable angina	281 (5.8)	252 (5.7)	29 (6.9)	0.374
Recent myocardial infarction	1901 (39.5)	1756 (39.9)	145 (34.7)	**<0.001**
Prior percutaneous coronary intervention	1325 (27.5)	1183 (26.9)	142 (34.0)	**0.002**
Left ventricular function				0.193
Good (<50%)	3694 (76.7)	3356 (76.3)	338 (80.7)	
Moderate (31–50%)	960 (19.9)	888 (20.2)	72 (17.2)	
Poor (21–30%)	122 (2.5)	116 (2.6)	6 (1.4)	
Very poor (<20%)	25 (0.5)	24 (0.5)	1 (0.2)	
Chronic lung disease	334 (6.9)	310 (7.1)	24 (5.7)	0.573
Pulmonary Hypertension	55 (1.2)	50 (1.1)	5 (1.2)	0.978
Poor mobility	99 (2.1)	91 (2.1)	8 (1.9)	0.888
Neurological dysfunction	113 (2.3)	102 (2.3)	11 (2.6)	0.817
Previous cerebral vascular accident	233 (4.8)	212 (4.8)	21 (5.0)	0.949
Dialysis	21 (0.4)	17 (0.4)	4 (1.0)	0.192
NYHA class				**0.010**
I	2850 (59.2)	2624 (59.7)	226 (54.1)	
II	1707 (35.5)	1549 (35.2)	158 (37.8)	
III	239 (5.0)	206 (4.7)	33 (7.9)	
IV	8 (0.2)	8 (0.2)	0	
CCS IV	266 (5.5)	240 (5.5)	26 (6.2)	0.594
Extend of CAD				
Single vessel disease			26 (6.2)	
Double vessel disease			104 (24.9)	
Triple vessel disease			288 (68.9)	
Left main disease			133 (31.8)	
Level of urgency				0.231
Elective	1955 (40.5)	1784 (40.4)	277 (66.3)	
Urgent	2595 (53.7)	2377 (53.8)	112 (26.8)	
Emergency	263 (5.4)	234 (5.3)	29 (6.9)	
Salvage	0	0	0	
EuroSCORE II	1.30 [0.88–2.10]	1.31 [0.88–2.10]	1.23 [0.85–1.91]	0.478
Preoperative hemoglobin (mmol/L)	8.8 [8.2–9.3]	8.8 [8.2–9.4]	8.7 [8.1–9.3]	0.102
Preoperative hematocrit	0.43 [0.40–0.45]	0.43 [0.40–0.45]	0.42 [0.40–0.45]	0.126
Preoperative creatinine (mL/min/1.72 m^2^)	86 [76–100]	86 [76–100]	86 [74–98]	0.764

NYHA, New York Heart Association; CCS, Canadian Cardiovascular Society. Significant *p*-values are formatted in bold.

**Table 2:** Perioperative and postoperative characteristics.

	Overall (N = 4814)	No Reintervention (N = 4396)	Reintervention (N = 418)	*p*-Value
Graft conduit				**<0.001**
Single arterial	251 (5.2)	214 (4.9)	37 (8.9)	
Single arterial + SVG	4022 (85.5)	3699 (84.1)	323 (77.3)	
Multi arterial + SVG	53 (1.1)	44 (1.0)	9 (2.2)	
Total arterial	388 (8.1)	349 (7.9)	39 (9.3)	
Total venous	100 (2.1)	90 (2.1)	10 (2.4)	
Number of distal anastomoses	3.3 ± 1.0	3.3 ± 1.0	3.1 ± 1.1	**<0.001**
Number of arterial grafts	1.3 ± 0.75	1.3 ± 0.7	1.3 ± 0.8	0.426
Number of venous grafts	2.0 ± 1.16	2.0 ± 1.2	1.8 ± 1.2	0.752
Off-pump	1107 (23.0)	983 (22.4)	124 (29.7)	**0.001**
Blood transfusion				
Red blood cells	1060 (22.0)	952 (21.6)	108 (25.8)	0.056
Blood plasm	173 (3.4)	156 (3.5)	17 (4.1)	0.205
Thrombocytes	366 (7.6)	334 (7.6)	32 (7.7)	0.239
Hospital stay (Days)	5.0 [4.0–6.0]	5.0 [4.0–6.0]	5.0 [4.0–7.0]	**<0.001**
Readmission ICU	100 (2.1)	80 (1.8)	20 (4.8)	**<0.001**
Ventilation > 24 h	90 (1.9)	73 (1.7)	17 (4.1)	**0.001**
Mortality				
In hospital	32 (0.7)	28 (0.6)	4 (1.0)	0.650
Overall	394 (8.2)	349 (7.9)	45 (10.8)	0.095

SVG, saphenous vein graft; ICU, Intensive care unit. Significant *p*-values are formatted in bold.

**Table 3:** Medication at hospital discharge.

	Reintervention Group (N = 418)
Antihyperlipidemic medicines	
Statins	327 (78.2)
Ezetimibe	10 (2.4)
PSCK9 inhibitor	3 (0.7)
Fibrate	1 (0.4)
Combination medications	32 (7.7)
Unknown/Hospital Death	14 (3.3)
Anticoagulation	
Acetylsalicylic acid	173 (41.4)
P2Y12 inhibitor	11 (2.6)
Dual antiplatelet therapy	137 (32.8)
DOAC	4 (1.0)
Vitamin K antagonists	1 (0.2)
Dual therapy *	31 (14.6)
Triple therapy **	16 (3.8)
None	2 (0.5)
Unknown/Hospital Death	14 (3.3)
ACE-inhibitors/ARB	
Yes	250 (59.8)
No	154 (36.8)
Unknown/Hospital Death	14 (3.3)
Beta-blockers	
Yes	378 (90.4)
No	26 (6.2)
Unknown/Hospital Death	14 (3.3)
Calcium channel blockers	
Yes	101 (24.2)
No	303 (72.5)
Unknown/Hospital Death	14 (3.3)
Diuretics	
Yes	321 (76.8)
No	82 (19.6)
Unknown/Hospital Death	14 (3.3)

PSCK9 inhibitor, proprotein convertase subtilisin/kexin type 9; DOAC, direct oral anticoagulants; ACE-inhibitor, Angiotensin Converting Enzyme inhibitor; ARB, Angiotensin Receptor Blocker; * Dual therapy: antiplatelet therapy and vitamin K antagonist or DOAC; ** Triple therapy: dual antiplatelet therapy and vitamin K antagonist or DOAC. Significant *p*-values are formatted in bold.

**Table 4:** Reintervention parameters.

	Reintervention Group (N = 418)
Reintervention type	
Coronary artery bypass grafting	40 (9.6)
Percutaneous coronary intervention	378 (90.4)
Reason intervention	
Progression disease	85 (20.3)
Graft failure	270 (64.6)
Incomplete revascularization	44 (10.5)
Combined	17 (4.1)
Unknown	2 (0.5)
Graft failure conduit (total used grafts)	(as percentage of total used grafts)
Left internal mammary artery (n = 4692)	111 (2.4)
Right internal mammary artery (n = 246)	8 (3.3)
Radial artery (n = 214)	13 (6.1)
Venous (n = 4175)	182 (4.4)
Unknown	2 (0.5)

**Table 5:** Univariable and multivariable Cox regression analysis for the occurrence of reintervention.

	Univariable Analysis			Multivariable Analysis		
Variable	Hazard Ratio	95% CI	*p*-Value	Hazard Ratio	95% CI	*p*-Value
Female gender	1.46	1.16–1.84	**0.001**	0.88	0.50–1.55	0.664
Age (year)	1.01	1.00–1.03	**0.011**	1.02	0.99–1.05	0.165
Height (cm)	0.98	0.97–0.99	**0.001**	1.02	0.99–1.05	0.151
Weight (kg)	0.99	0.98–1.00	**0.001**	0.98	0.97–1.00	**0.044**
Diabetes	1.01	1.00–1.02	**0.076**	1.02	1.00–1.04	**0.011**
No treatment						
Diet						
Oral medication						
Insulin						
Peripheral vascular disease	1.15	0.90–1.48	0.259			
Atrial fibrillation	1.03	0.99–1.07	0.132			
Paroxysmal						
Non-paroxysmal						
Unstable angina	1.16	0.80–1.70	0.429			
Recent myocardial infarction	1.13	0.93–1.39	0.222			
Prior percutaneous coronary intervention	0.90	0.73–1.10	0.290			
Left ventricular function						
Good (<50%)	1.50	0.21–10.69	0.686			
Moderate (31–50%)	2.01	0.28–14.45	0.490			
Poor (21–30%)	1.11	0.13–9.22	0.925			
Very poor (<20%)	6.29	0.39–101.35	0.195			
Chronic lung disease	1.26	0.83–1.91	0.270			
Pulmonary Hypertension	1.05	0.99–1.11	0.130			
Poor mobility	1.87	0.93–3.78	**0.080**	2.79	0.21–37.65	0.440
Neurological dysfunction	1.88	1.03–3.43	**0.040**	1.48	0.70–3.12	0.300
Previous cerebral vascular accident						
Dialysis	0.98	0.36–2.62	0.965			
NYHA class	1.05	0.91–1.22	0.510			
I						
II						
III						
IV						
CCS IV	1.35	0.91–2.01	0.140			
Level of urgency	1.01	0.99–1.03	0.191			
Elective						
Urgent						
Emergency						
Salvage						
Euroscore II	1.05	1.01–1.09	**0.015**	0.94	0.77–1.14	0.517
Preoperative hemoglobin (mmol/L)	0.90	0.81–1.01	**0.062**	1.00	0.79–1.26	0.985
Preoperative hematocrit	0.56	0.33–0.96	**0.035**	0.64	0.32–1.28	0.208
Preoperative creatinine (mL/min/1.72 m^2^)	1.00	0.99–1.00	0.641			
ECC use	1.00	0.99–1.01	0.665			
Graft conduit						
Single arterial	1.79	1.28–2.52	**<0.001**	2.26	1.31–3.91	**0.003**
Single arterial + SVG	0.97	0.77–1.22	0.793			
Multi arterial + SVG	1.03	0.53–2.00	0.928			
Total Arterial	0.75	0.54–1.05	**0.090**	1.48	0.70–3.12	0.300
Total Venous	0.82	0.44–1.53	0.527			
Number of distal anastomoses	1.03	0.93–1.13	0.588			
Arterial grafts	1.00	0.88–1.13	0.960			
Venous grafts	1.02	0.94–1.11	0.613			
Blood transfusion						
Red blood cells	1.50	1.21–1.87	**<0.001**	1.11	0.71–1.74	0.642
Blood plasm	1.81	1.09–2.99	**0.022**	1.42	0.73–2.77	0.303
Thrombocytes	1.09	0.74–1.62	0.651			
Mortality						
In hospital	19.15	6.78–54.12	**<0.001**	1.02	0.98–1.06	0.260
Overall	2.09	1.53–2.87	**<0.001**	1.50	0.77–2.95	0.237
Hospital stay (Days)	1.01	1.00–1.03	**0.026**	1.02	0.98–1.06	0.260
Readmission ICU	1.88	1.19–2.95	**0.006**	0.74	0.32–1.69	0.472
Ventilation > 24 h	3.02	1.85–4.93	**<0.001**	4.61	1.85–11.51	**0.001**

NYHA, New York Heart Association; CCS, Canadian Cardiovascular Society; ECC, Extracorporeal circulation; SVG, Saphenous vein graft. Significant *p*-values are formatted in bold.

## Data Availability

Data will be made available by the corresponding author upon reasonable request.

## Abbreviations

The following abbreviations are used in this manuscript:

ACS	Acute coronary syndrome
ACE	Angiotensin Converting Enzyme
ARB	Angiotensin Receptor Blocker
CAD	Coronary artery disease
CABG	Coronary artery bypass grafting
CAG	Coronary angiogram
CCS	Canadian Cardiovascular Society
CVA	Cerebrovascular accident
DOAC	Direct Oral Anticoagulants
ECC	Extracorporeal circulation
Endo-CAB	Endoscopic Coronary Artery Bypass
ICU	Intensive Care Unit
IQR	Interquartile range
LIMA	Left internal mammary artery
LVEF	Left ventricle ejection fraction
NHR	Netherlands Heart Registration
NYHA	New York Heart Association
PCI	Percutaneous coronary intervention
PSCK9	Proprotein Convertase Subtilisin/Kexin type 9
RIMA	Right internal mammary artery
SD	Standard deviation
SMD	Standard mean differences
SVG	Saphenous vein graft
TVR	Target vessel revascularization
